# Permafrost thaw has enlarged the burned area proportion from naturally-ignited wildfires in Arctic-boreal regions

**DOI:** 10.1093/nsr/nwag515

**Published:** 2026-08-20

**Authors:** Lei Cai, Weiyu Shi, Qiang Zhang, Peter B Reich, Pierre Gentine, Fang Li, Wenbing Ouyang, Xiankui Li, Vladimir A Alexeev, Alexander Shiklomanov, Jie Cao, Ruowen Yang

**Affiliations:** Yunnan Key Laboratory of Meteorological Disasters and Climate Resources in the Greater Mekong Subregion, Yunnan University, Kunming 650500, China; Chongqing Jinfo Mountain Karst Ecosystem National Observation and Research Station, School of Geographical Sciences, Southwest University, Chongqing 400715, China; Ministry of Education Key Laboratory for Earth System Modeling, Department of Earth System Science, Tsinghua University, Beijing 100084, China; Institute for Global Change Biology, University of Michigan, Ann Arbor, MI 48109, USA; Department of Forest Resources, University of Minnesota, St Paul, MN 55108, USA; Department of Earth and Environmental Engineering, Columbia University, New York City, NY 10027, USA; Institute of Atmospheric Physics, Chinese Academy of Sciences, Beijing 100029, China; Yunnan Key Laboratory of Meteorological Disasters and Climate Resources in the Greater Mekong Subregion, Yunnan University, Kunming 650500, China; Institute of Atmospheric Physics, Chinese Academy of Sciences, Beijing 100029, China; Yunnan Key Laboratory of Meteorological Disasters and Climate Resources in the Greater Mekong Subregion, Yunnan University, Kunming 650500, China; International Arctic Research Center, University of Alaska Fairbanks, Fairbanks, AK 99775, USA; School of Marine Science and Ocean Engineering, University of New Hampshire, Durham, NH 03824, USA; Yunnan Key Laboratory of Meteorological Disasters and Climate Resources in the Greater Mekong Subregion, Yunnan University, Kunming 650500, China; Yunnan Key Laboratory of Meteorological Disasters and Climate Resources in the Greater Mekong Subregion, Yunnan University, Kunming 650500, China

**Keywords:** wildfires, permafrost thaw, Arctic warming, fire ignition causes

## Abstract

Recent studies revealed a simultaneous increase in Arctic-boreal wildfires and permafrost thaw since 2000. Despite the agreement that wildfires accelerate permafrost thaw, it remains unclear whether permafrost thaw makes the landscapes more prone to wildfires. Our study developed a model-based approach to disentangle wildfires ignited naturally and anthropogenically. The historical simulation demonstrates an abrupt increase in the proportion of burned area by naturally-ignited wildfires that occurred around 2010 in the Arctic-boreal regions. The increase in burned area proportion results from the shallow-layer soil drying due to permafrost thaw. Model results and observations verified rapid permafrost thaw in the early 2000s, followed by soil drying lagged by 4.29 ± 1.52 years. The shallow soil drying that can enhance fuel combustibility is more pronounced in permafrost regions dominated by natural wildfires. Our study demonstrated that permafrost thaw has become a critical driver of Arctic-boreal wildfires, especially for the naturally-ignited fires that have a larger burned area on average per event in permafrost regions. The resulting positive feedback, namely, permafrost thaw promoting more fires, and fires further accelerating thaw, could foreshadow profound climate repercussions.

## Teaser

A larger proportion of burned area have been caused by naturally-ignited wildfires in the Arctic-boreal regions since 2010, being driven by accelerated permafrost thaw in the early 2000s and the corresponding shallow soil drying.

## INTRODUCTION

The Arctic-boreal area has undergone dramatic warming, earning the name Arctic amplification [1], and the permafrost thaw and wildfire intensification are known as the impacts of warming [2]. The burning of both vegetation and soil organic matter from wildfires decreases surface albedo and enhances the thermal and hydrological conductance of the ground surface, which accelerates ground temperature increase and then permafrost thaw [2,3]. On the other hand, some studies have suggested that the thawing of permafrost enhances fuel loads and their combustibility, therefore promoting wildfires [4–6].

Since 2000, the burned area from wildfires has encountered a decline characterized by a shrinkage in savanna fires in tropical areas [7], although this may have increased since the mega fires in Canada in 2023 [8]. Wildfires in the Arctic-boreal regions, however, have shown an increasing trend in both burned area and fire emissions [7,9]. Specifically, Canada and Alaska together have experienced a nearly 40% increase in burned areas in the 2010s compared to the 2000s [9]. Wildfires over permafrost are generally considered uncommon, although many have burned across the permafrost-rich area in recent years, especially after 2020 [10,11]. For future wildfire projections, Earth System Model projections under the Shared Socioeconomic Pathway 3-7.0 (SSP3-7.0) scenario have suggested an abrupt wildfire intensification starting around 2050, due to permafrost thaw and corresponding soil drying [12]. From the perspective of the recent Arctic-boreal wildfire enhancements, it remains unclear whether permafrost thaw and the corresponding thermal and hydrological impacts have an effect.

Wildfires can be categorized by ignition sources into natural and anthropogenic fires. In Arctic-boreal regions, most natural wildfires are ignited by lightning strikes. Some studies have attributed the Arctic-boreal wildfire enhancements to the increases in lightning frequency [13,14]. Natural wildfires differ from those ignited by humans (anthropogenic wildfires) in having more distinctive seasonality and higher sensitivity to environmental changes [13,15]. Exploring the effect of permafrost motivates the necessity to investigate wildfire regimes with their ignition sources distinguished. However, the wildfire ignition source is technically difficult to observe and, therefore, absent from most datasets. There are barely any fire type distribution datasets currently available, and they only record the spatial patterns rather than the temporal variabilities of wildfire types [14,16–18]. Despite the current process-based fire models respectively parameterizing natural and anthropogenic fires with different schemes, considerable model biases, especially in the Arctic-boreal area [19,20], limit the understanding of the relationship between wildfires and permafrost changes.

In this study, we explored whether and how thawing permafrost has driven the spatiotemporal variations of wildfires in the Arctic-boreal regions by involving specific modifications to the permafrost thawing processes and fire ignition parameterizations in the Community Land Model, version 5 (CLM5) [21]. We incorporated sub-grid-scale excess ground ice and its melting processes [22] to better represent the thermal and hydrological responses to permafrost thaw (see the supplementary materials for more details). We subsequently optimized both the natural and anthropogenic fire ignition parameters independently for each model grid cell (Fig. S1) by conducting an ensemble of sensitivity simulations and addressing grid-scale parameter calibrations with satellite-observed wildfire occurrences as the reference (see ‘Fire model modification’ in the supplementary materials). The modified CLM5 model was improved at reproducing the climatology of wildfire occurrences globally (Fig. S2), exporting the fields of occurrence and the burned area of natural and anthropogenic wildfires disentangled from each other. The spatiotemporal distributions of Arctic-boreal wildfires from 2000 to 2022 were compared with the thermal and hydrological regimes of Arctic-boreal permafrost and their changes due to permafrost thaw. We explored the evidence from observations to verify the process discovered from model results and to elucidate the role of permafrost thaw in the current Arctic-boreal wildfire regimes.

## RESULTS AND DISCUSSION

### Shift in the burned area proportion of naturally-ignited Arctic-boreal wildfires

Following modification, the CLM5 model produced a spatial pattern on the percentage of burned area from natural wildfires, presenting a consistency in general with the fire attribution map processed from machine learning techniques [14]. The spatial distribution was also in agreement with observations in some regions, including the United States, Canada, parts of Siberia, and scattered regions in Australia and Spain, where ignition-type observations are available [17,18] (Fig. S3; see the supplementary materials for more details). Over the Arctic-boreal regions defined as the latitudes north of 60°N in this study, natural wildfires dominated in northern Canada and a large part of Siberia. Most of these regions were sparsely populated, and the fires were categorized by peat fires modeled with a separate parameterization scheme (Fig. 1a; additional details regarding wildfire parameterizations in the CLM5 are provided in the Fire model modification section in the supplementary materials). From the nature of peat fires, our algorithm regarded all the modeled peat fires in the Arctic-boreal regions as naturally ignited. Northern Europe, including Scandinavia, and scattered regions in Alaska, were found to be dominated by anthropogenic fires. A mix of natural and anthropogenic wildfires was distributed in central and eastern Siberia (Fig. 1a). Furthermore, involving the uncertainties in fire ignition parameters does not bring distinctive difference in the spatial pattern of fire attribution in the Arctic-boreal regions (Fig. S4). Comparing with the Global Fire Emission Database 5 (GFED5) [23], our model captured a similar interannual variability of burned area in Arctic-boreal regions with a Pearson correlation coefficient of 0.54 and *P*-value of 0.016 (Fig. 1b). The modeled burned area also shared a similar spatial pattern in the annual mean burned area to that in the GFED5 (Fig. S5), verifying the improved model performance from our modifications. The upsurge of Arctic-boreal wildfires in 2021 [10,12] also appeared in the modeled temporal variation, although the variance was not as high as in the observations.

**Figure 1.**
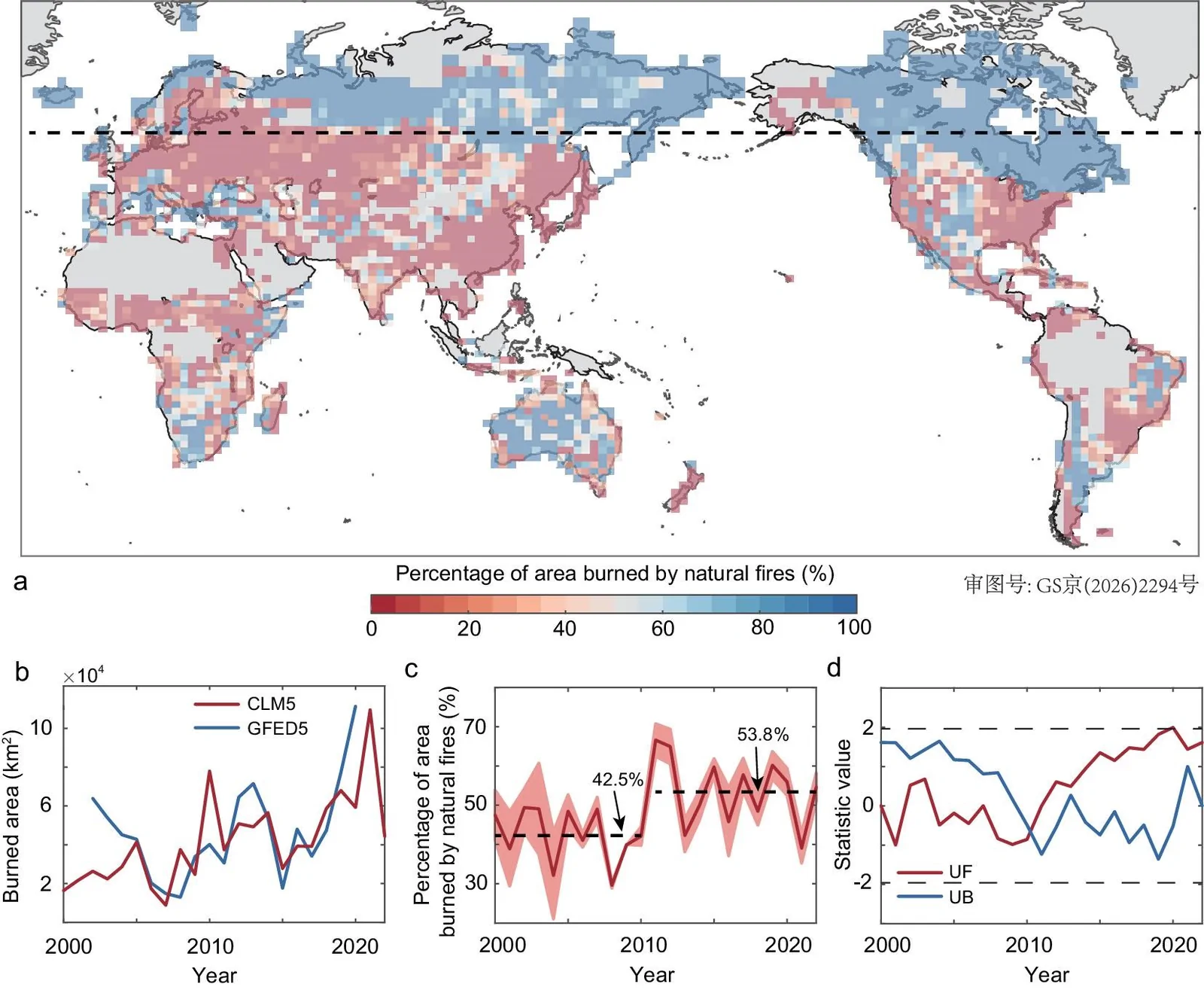
Modeled spatiotemporal distributions of wildfire type attributions. (a) Spatial pattern of the modeled percentage of burned area by natural wildfires by our modified CLM5 model. The percentage was calculated only in the grids with one or more fire events modeled annually. (b) Timeseries of Arctic-boreal burned area (km^2^) calculated from the CLM5 model results (red line) and the GFED5 data (blue line). The Arctic-boreal area was defined as 60°N and poleward, and it was delimited by the black dashed line in (a). (c) Time series of the proportion (%) of burned area from natural fires (red line). The red shading in (c) indicated the uncertainty calculated from simulations involving uncertainties of fire ignition parameters. The dashed lines indicated the mean value in the periods of 2000–2010 and 2011–2022. (d) The sequential Mann-Kendall test results on the timeseries of burned area proportion by natural fires. UF (red line) and UB (blue line) are the test statistics calculated forward and backward from the time series of the burned area percentage from natural wildfires, respectively. Their intersection(s) represent the specific year(s) after which the time series abruptly changed. The intersection lying between the two black dashed lines indicated the point of abrupt change passing the 95% confidence level.

Our modeled results suggested a shift in the percentage of burned area from natural fires in the Arctic-boreal regions around 2010. From 2000 to 2010, natural wildfires were responsible for an average of 42.5% of burned area in Arctic-boreal regions, while the average proportion increased to 53.8% from 2011 to 2022. The difference between the means was statistically significant at the confidence level of 95% by the student’s *t*-test (Fig. 1c). In this way, natural wildfires were modeled to be the prevailing wildfire type in Arctic-boreal regions after 2010. Regarding the changing point of 2010, the sequential Mann-Kendall test verified its statistical significance at the 95% confidence level (Fig. 1d). The sequences UF and UB intersected between 2010 and 2011, and the values of UF and UB at the intersection were within ±1.96, suggesting a statistical significance of the shift happening between 2010 and 2011 (Fig. 1d; additional details of the significance test can be found in the Sequential Mann-Kendall test in the supplementary materials). Such a shift was initiated more than 10 years in advance compared to the record-breaking wildfire activities after 2020 in the Arctic-boreal region [7,9]. Both the GFED5 and our model results showed a similar pattern in annual mean burned area differences between the periods of 2011–2022 and 2000–2010 (Fig. S6a and b). Burned area increased in western and central Siberia and northern Canada, while decreases in burned area were found in eastern Siberia and Alaska. The percentage of burned area changes is typically greater than 50% (Fig. S6c and d). In correspondence to the fire attribution map, the burned area increases in northern Canada and western Siberia were attributed to the change in natural fires, while the burned area decreases in Alaska were attributed to the change in anthropogenic fires (Fig. S7). Central Siberia encountered an increase in burned area from both natural and anthropogenic fires, but the magnitude of the increase was greater for natural fires in this area. It was verified that the changes in the Arctic-boreal wildfire regimes, especially the abrupt increase in the proportion of natural fires, were not homogeneous throughout the Arctic-boreal area, but a shift in spatial distributions. The regional climate change impacts were therefore worthwhile being analyzed to unveil the mechanism to explain the abrupt increase in natural fires.

### Divergent wildfire responses depending on soil hydrology and fire types

According to the fire model, wildfires are simulated only when the 0–17 cm soil temperature is greater than 0°C [19], defining the fire season in a year. In this way, most Arctic-boreal wildfires only exist during summer. An examination of the changes in temperature and precipitation in 2011–2022 compared to 2000–2010, averaged by the model fire season, suggested a slight increase in surface air temperature and a decrease in precipitation (Fig. S8). The temperature change pattern did not correspond spatially to the wildfire change patterns. The decreases in precipitation were typically less than 10%, which could hardly account for such a great increase in burned area after 2010. Meanwhile, such an inconsistency implied that other than the climatic changes in temperature and precipitation, there must be other contributing factors controlling the abrupt shift in the percentage of natural fires in the Arctic-boreal regions.

The fire model in this study [19] parameterized the moisture conditions of both the atmosphere and soil as suppression factors of wildfire ignition and spread. So theoretically, drier air and shallow-layer soil should result in a larger burned area (see ‘land surface modeling’ in the supplementary materials). Specifically, the moisture condition in shallow soil layers has a dominant role in determining fuel combustibility compared to that in deep soil layers. The algorithms of fire rating systems in countries including Canada, the United States, and Australia apply shallow-layer soil moisture to indicate fuel moisture and combustibility [24]. Climatologically, multiple datasets recorded an accelerated warming in the boreal-Arctic since 2000 [25,26]. Specifically, the ERA5 reanalysis data [27] recorded a 0.69°C/decade increase in surface air temperature over the Arctic-boreal land (Fig. S9a), while precipitation did not reveal a significant increase (Fig. S9b). Considering the greater drying power of a warmer atmosphere, evapotranspiration almost certainly increased, and with stable precipitation, which almost certainly led to more frequent periods of low soil moisture. Forced by the ERA5, the CLM5 model consequently presented a consistent drying trend after 2007, represented by the Climatic Water Deficit (CWD), corresponding well in time with the shift in natural wildfires around 2010 (Fig. 2a). The calculation of CWD followed the methods established by Abatzoglou *et al.* [28] as the amount of water (mm) from precipitation minus that from potential evapotranspiration.

**Figure 2.**
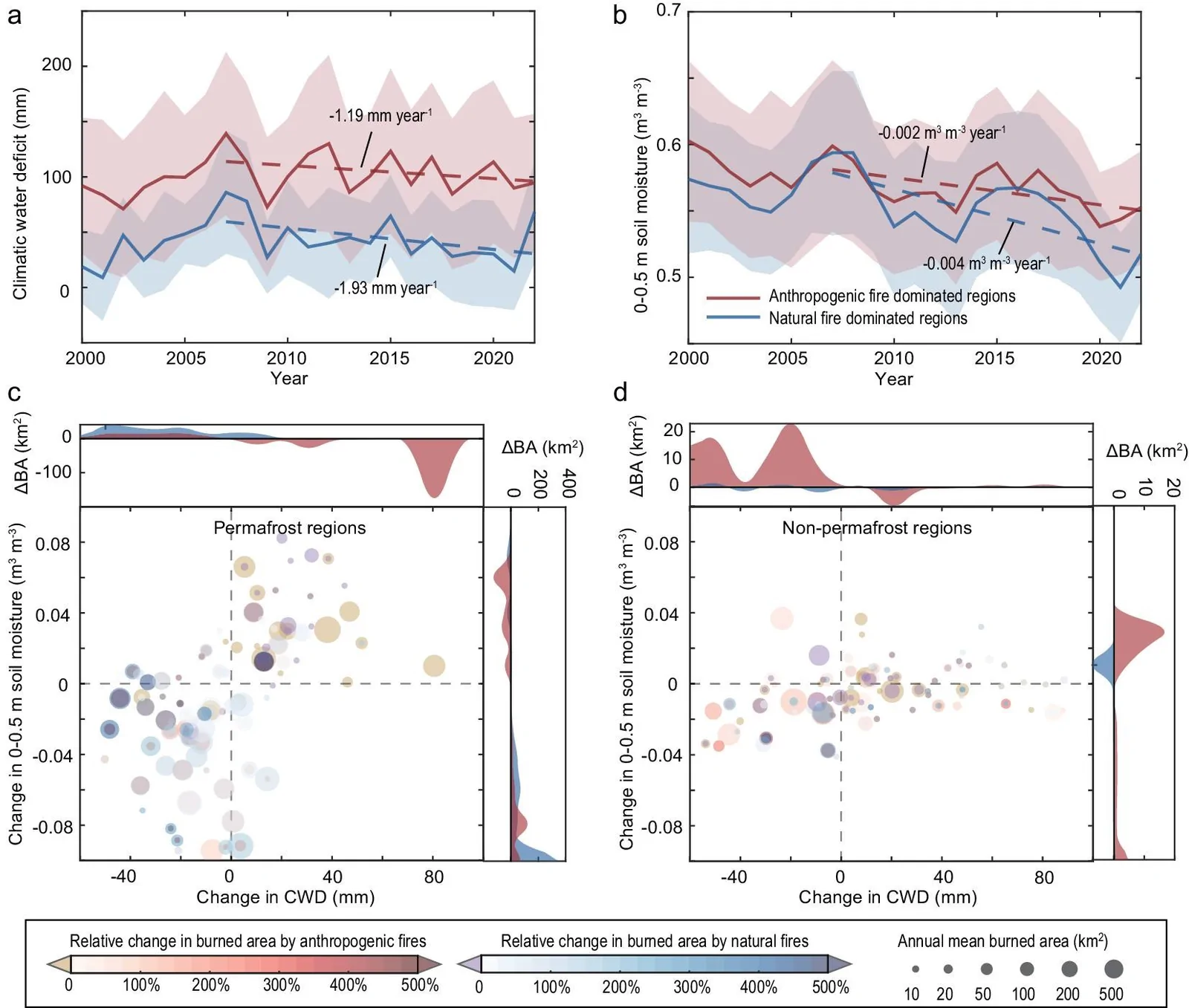
Wildfire responses to changes in CWD and soil moisture. The CWD was calculated as the difference between precipitation and potential evapotranspiration. Time series of (a) CWD and (b) soil moisture averaged at depths of 0–0.5 m in regions dominated by anthropogenic (red lines) and natural (blue lines) fires, respectively. Relative change (%) in burned area in response to the changes in CWD and soil moisture between the two periods of 2011–2022 and 2000–2010 in (c) permafrost regions and (d) non-permafrost regions. The dot color indicated the relative differences in burned area caused by natural and anthropogenic fires, respectively, in the same period. The negative difference in burned area is shown in purple and brown for natural and anthropogenic fires, respectively. The size of the dots indicates the mean annual burned area over 2000–2022. The probability density functions on the boundary show the magnitude change (km^2^) of burned area (ΔBA), respectively, by natural (blue shaded) and anthropogenic (red shaded) fires.

Our model results suggested that the Arctic-boreal regions dominated by natural fires had a drier climate represented by the lower CWD climatology than those dominated by anthropogenic fires (Fig. 2a). This divergence was consistent with the precipitation climatology that was lower in northern Canada and Siberia than in Scandinavia and Western Europe [29]. Furthermore, regions dominated by natural wildfires suffered not only from a drier climate but also a greater trend towards drying for both atmosphere and soil (Fig. 2b). The CWD and 0–0.5 m soil moisture decreased at rates of −1.19 mm year^−^^1^ and −2 × 10^−3^ m^3^ m^−^^3^ year^−^^1^, respectively, for regions dominated by anthropogenic wildfires. Regions with natural fires, in comparison, had decreasing trends of −1.93 mm year^−^^1^ for CWD and −4 × 10^−3^ m^3^ m^−^^3^ year^−^^1^ for 0–0.5 m soil moisture. Both the extra drying and its starting point in the year 2007 corresponded well with the increase in the ratio of Arctic-boreal natural wildfires.

Note that the regions with the greatest increase in natural fires, including northern Canada and western and central Siberia, were within the permafrost area, which was defined as the regions where the modeled active layer depth was less than 9.85 m by the end of the spin-up (Fig. S10). A similar shift in the percentage of wildfires that occurred over Arctic-boreal permafrost was also present around 2010. In 2000–2010, 81.0 ± 12.8% of the burned area of Arctic-boreal wildfires occurred over permafrost, while the percentage increased to 92.4 ± 6.7% in 2011–2022 (Fig. S11a). There was also an abrupt change around 2010 in the time series of the percentage of burned area in permafrost regions verified by the sequential Mann-Kendall test (Fig. S11b).

Comparing the modeled CWDs and soil moisture conditions between the two periods of 2000–2010 and 2011–2022, averaged in the regions with differing dominances of wildfires, the modeled results embodied divergent responses of burned area increase in and out of permafrost regions. We specifically focused on the grid cells with decreases in CWD and soil moisture that were supposed to increase burned area according to the parameterization. Within the Arctic-boreal permafrost, the modeled natural wildfires burned 8671.6 ± 67.5 km^2^ more area annually from 2011 to 2022 than from 2000 to 2010, and the burned area increase from anthropogenic wildfires (507.9 ± 68.9 km^2^) was 94.1% smaller (Fig. 2c). The modeled changes in CWD and soil moisture co-varied significantly. Low CWDs (i.e. a large negative value) typically corresponded to low soil moisture, which together corresponded to burned area increases. More importantly, the decreases in CWD and soil moisture led to greater burned area increases from natural wildfires (123.0 ± 67.7 km^2^) than from anthropogenic fires (65.4 ± 45.9 km^2^) for each grid cell on average. Meanwhile, the increases in CWD and soil moisture also resulted in a smaller decrease in burned area from natural wildfires (4.3 ± 45.1 km^2^) than from anthropogenic wildfires (−147.9 ± 193.1 km^2^) for each grid cell on average. Additional statistics further indicated that natural fires result in a larger burned area on average after each ignition than anthropogenic fires do, which might originate from the lack of wildfire suppression in sparsely populated regions where natural fires were the dominant fire type parameterized by the fire model (Fig. S12). A more favorable environmental background after 2010 within the permafrost would therefore lead to greater increases in burned area from natural than anthropogenic wildfires, eventually represented by a larger proportion of burned area from natural fires (Fig. 1c).

The Arctic-boreal regions without permafrost showed smaller burned area increases than those within permafrost, and most of this increase was due to anthropogenic rather than natural wildfires. In 2011–2022, the burned area from anthropogenic fires increased by 1134.6 ± 40.8 km^2^ while that from natural fires decreased by 474.6 ± 17.2 km^2^ (Fig. 2d). More importantly, these regions did not present any explicit changes in soil moisture, so there weren’t any covariations found between CWD and soil moisture responses. The magnitudes of change in CWD were similar between the regions in and out of the permafrost, but failed to result in any consistent changes in wildfires, either natural or anthropogenic ones (Fig. 2d). These model-based wildfire dependencies demonstrated that the change in the Arctic-boreal permafrost could result in a critical difference in the environmental background that determines wildfire dynamics. The shallow soil moisture presented the most divergent responses in and out of the permafrost area, implying that the change in permafrost soil condition under climate change could make the critical difference.

### Correspondence in the timeline of permafrost thaw, soil drying, and wildfire increase

Permafrost consists of the soil that has been frozen for 24 consecutive months or longer, which is covered by a layer of soil on top that is subjected to freeze and thaw annually, namely the active layer [30]. The annual maximum of active layer thickness, called the annual thaw depth, serves as an important measurement of permafrost thermal state [31]. Permafrost is typically low in hydraulic conductivity because soil water present in the form of ice fills the pore spaces of the soil [32]. The unfrozen soil water in warmer months tends to accumulate above the active layer, making a permafrost water table. In this way, the moisture condition in shallow soil layers is more sensitive to the climatic background in permafrost than out of permafrost, which explains the divergent co-variations of CWD and 0–0.5 m soil moisture in and out of the Arctic-boreal permafrost (Fig. 2c and d). When the permafrost temperature rises above freezing, the soil ice melts, and hydraulic conductivity increases [33]. Due to the substantial difference in soil hydraulic conductivity between the active layer and permafrost beneath, liquid water tends to percolate and accumulate in a thin layer above the permafrost table [33,34], therefore leaving less moisture in the shallow soil layers and forming a favorable condition for wildfires. The modeled changes in water fluxes verified the mechanism above that wildfire increase was consistently associated with 0–0.5 m soil drying (Fig. 3). For permafrost regions with increased wildfires after 2010, changes in percolation led to a relative contribution of 91.0% in soil drying. In comparison, the increase in evapotranspiration due to the rising temperature (32.3%) was just above one-third in relative contribution. Permafrost regions with wildfire decreases are typically associated with decreases in temperature and increases in precipitation during fire seasons after 2010, protecting permafrost from thawing and therefore resulting in percolation decreases (Figs S6 and S8). The enhanced precipitation as the water source led to higher moisture content in shallow soil layers. Out of the permafrost regions, shallow soil moisture change was dominantly controlled by either evapotranspiration relative to temperature changes, or atmospheric water sources relative to precipitation changes. It suggested that over the Arctic-boreal regions, percolation enhancement following permafrost thaw makes the dominant contribution to shallow soil drying within permafrost regions, while the atmospheric background changes dominantly controlled shallow soil moisture change outside the permafrost regions.

**Figure 3.**
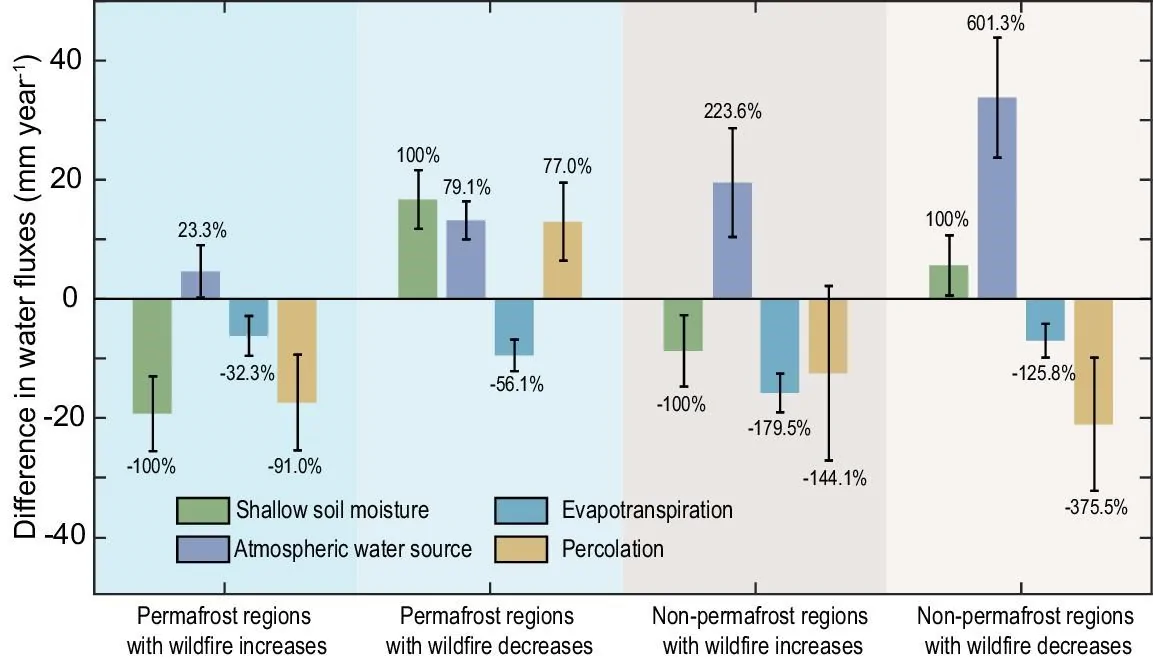
The changes in the water fluxes between 2011–2022 and 2000–2010 under different permafrost and wildfire regimes. The Arctic-boreal regions were divided into four different sub-categories depending on the permafrost regimes and wildfire changes. Shallow soil moisture was defined as that in soil layers at the depths of 0–0.5 m. The atmospheric water source was calculated from total precipitation, with surface runoff and snow sublimation excluded. The water flux at the soil depth of 0.5 m was regarded as the percolation. The relative contribution was calculated based on the changes in shallow soil moisture. Positive differences and relative contributions indicated more water was imported into shallow soil layers, and negative ones indicated more water was exported from shallow soil layers. For regions with shallow soil moisture increase, the relative contributions of atmospheric water source, evapotranspiration, and percolation added up to 100% in total. For regions with shallow soil moisture decrease, these relative contributions added up to −100% in total. Since the contributions of different factors partially canceled each other out, some relative contributions could possibly be higher than 100% or lower than −100%.

An idealized experiment was forced by the cycling atmospheric forcing without any interannual variations (see the supplementary materials for more details about the idealized experiments) to examine the soil moisture responses to active layer thickening without the disturbances of the transient climate background changes. A single-grid-cell examination based on the idealized experiments verified that the decreases in 0–0.5 m soil moisture coincided in time with wildfire increases, which consistently followed active layer deepening with a lag (Fig. S13a–d). The modeled lag from active layer deepening to shallow soil drying could be multiple years because the percolation was restricted by the freezing-thawing nature of permafrost soil in the active layer. Globally, grid cells with a lower annual mean temperature and a thinner active layer were associated with a longer lag time. The permafrost in a colder environment typically has a slow percolation rate because water is in the form of ice and therefore remains impeded for a longer period of the year. The lag time was 2.96 ± 2.04 years on average for the idealized simulation (Fig. S13e and f).

When forced by the real-time atmospheric forcing, the CLM5 model captured an acceleration of active layer deepening starting around 2004 over the Arctic-boreal permafrost, followed by substantial 0–0.5 m soil drying starting from 2010 (Fig. 4a). The modeled active layer depth increased from about 1 m in 2000 to about 1.4 m in 2020, and the soil layer with the drying anomaly thickened correspondingly. Near the thickness of the active layer, the negative anomaly of soil moisture turned into positive (Fig. 4a). The modeled soil moisture anomalies were limited within the upper layers of soil, with negligible anomalies below 4 m in depth (Fig. S14). The shallow soil drying started to be apparent when the active layer deepening became 0.2 m or more. A maximum of nearly 7% of soil moisture decrease corresponds to the 0.4–0.5 m active layer deepening for the Arctic-boreal permafrost (Fig. 4b). The soil moisture responses could differ substantially from permafrost thaw. A range from 30% drying to nearly 20% wetting for 0–0.5 m soil moisture change occurred across the Arctic-boreal permafrost compared to soil moisture in 2000 (Fig. 4b). But overall, the Arctic-boreal permafrost presented drying outweighing wetting.

**Figure 4.**
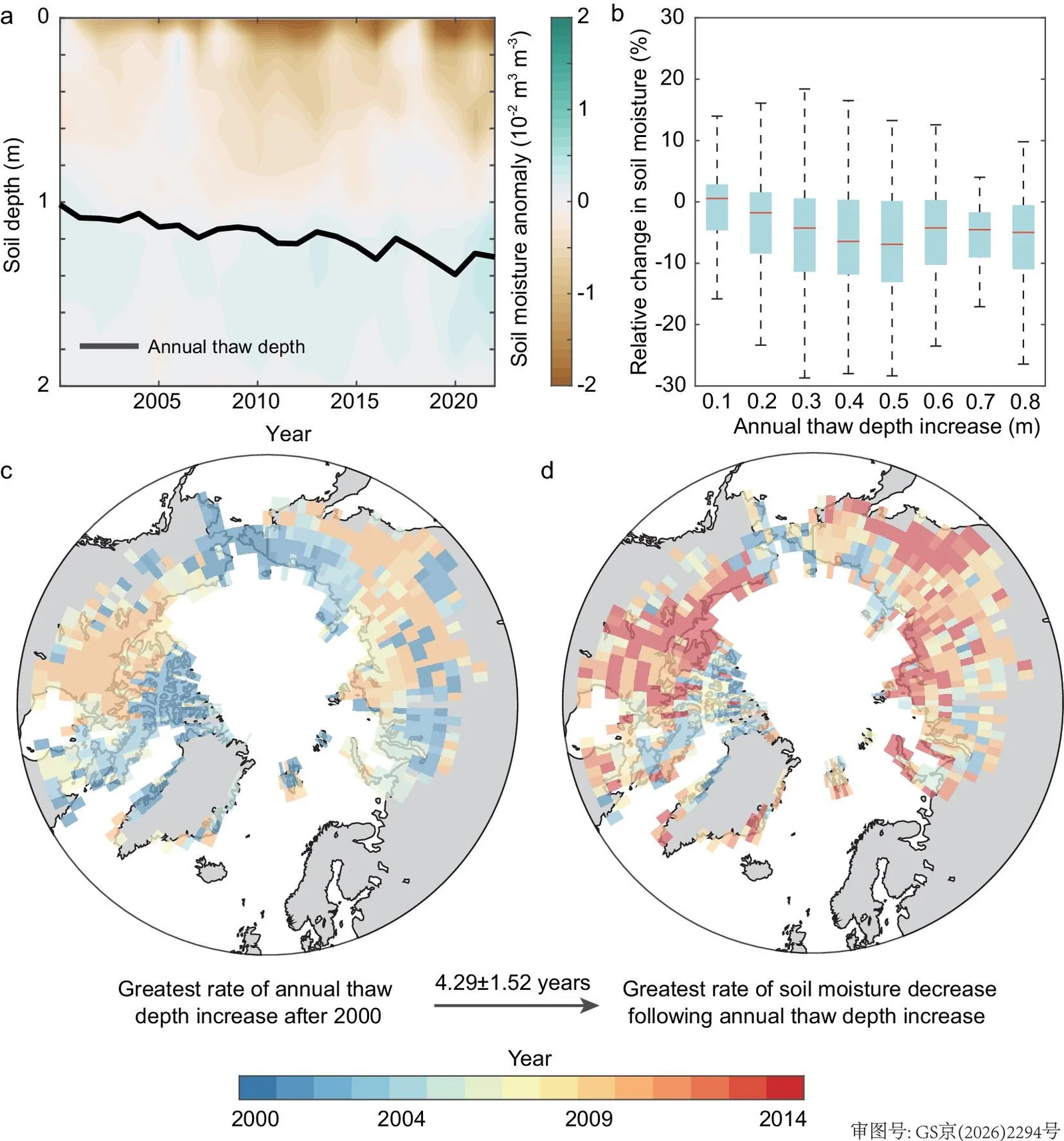
Modeled soil hydrological responses to permafrost thaw. (a) Soil moisture anomalies in the depth of 0–2 m compared to the last year of spin-up for the soil layers. Anomalies are calculated with the last year of spin-up as a reference for each corresponding soil layer. The black line indicates the annual thaw depth from 2000 to 2022, averaged over the Arctic-boreal permafrost. (b) Boxplot of the relative change (%) in 0–0.5 m soil moisture for different magnitudes of annual thaw depth increases compared to that in 2000. (c) Spatial distributions of the year with the greatest increasing rate of annual thaw depth and (d) with the following greatest decreasing rate of 0–0.5 m soil moisture. The lag time from the annual thaw depth increase to the soil moisture decrease was on average 4.29 ± 1.52 years. Some grid cells in (c) disappeared in (d). These cells exhibited a soil moisture increase rather than a decrease following an annual thaw depth increase.

The lag time from active layer thickening to soil moisture decrease is determined by how fast the water percolates down, which was affected by local climate, soil texture, moisture conditions, local topography, etc. [35–37]. The formation of inundated land after excess ice melt and surface subsidence could result in near-surface soil saturated with water. Spatially, the annual thaw depth thickening is simulated to accelerate mostly between 2003 and 2007 in the Arctic-boreal permafrost (Fig. 4c), followed by the acceleration of 0–0.5 m soil moisture decrease that happened around 2008 to 2012 (Fig. 4d). The years when the acceleration of annual thaw depth increase and soil moisture decrease happened were calculated from the maxima and minima of the second derivatives of the time series, respectively, for each grid cell. For parts of northern Alaska and eastern Siberia, the modeled annual thaw depth was the earliest to accelerate (from 2000 to 2003), where the soil moisture was found to increase instead of decrease as the response. These regions were rich in excess ground ice in permafrost according to the permafrost map [38], which worked as the excess ice input data in our simulations. The melt of excess ice tended to tentatively increase soil moisture in the early thaw stage [39,40], which explained the small increase in soil moisture within 0–0.1 m active layer deepening (Fig. 4b), as well as the soil moisture increase before its decrease following active layer deepening in the idealized simulation (Fig. S13b). The average lag time across the Arctic-boreal permafrost was 4.29 ± 1.52 years, which is slightly longer than that in the idealized simulation. This is because the precipitation amount kept increasing until 2007, therefore, tentatively compensating the water loss from infiltration and postponing the shallow soil drying after active layer thickening.

The thermal and hydrological changes from permafrost degradation, as well as their timing, were confirmed by multiple observational datasets. The annual thaw depths from 195 sites from the circumpolar active layer monitoring network (CALM) [41] recorded a rapid thickening of the active layer over the Arctic-boreal area from 2000 to 2010 (Fig. 5a). The increases in annual thaw depth from 2000 to 2010 accounted for 62% of the increase from 1990 to 2022 (Fig. 5b). In terms of soil moisture observations, 68 sites over continuous and discontinuous permafrost in Alaska and Canada from the International Soil Moisture Network (ISMN) [42] presented consistent decreases since 2003 (Fig. 5c and d). The soil moisture values measured at depths of 0.05 and 0.5 m decreased by 37% and 20%, respectively, for the period of 2011–2022 compared to 2003–2010. Meanwhile, soil moisture measured in 39 ISMN sites over sporadic permafrost or non-permafrost in Nordic countries barely presented any explicit decreases since 2003 (Fig. 5c and d). These non-permafrost regions had a much deeper water table [43] compared to the permafrost regions. The hydraulic conductivity of shallow soil, therefore, barely changed from warming, leading to negligible variations in soil moisture conditions.

**Figure 5.**
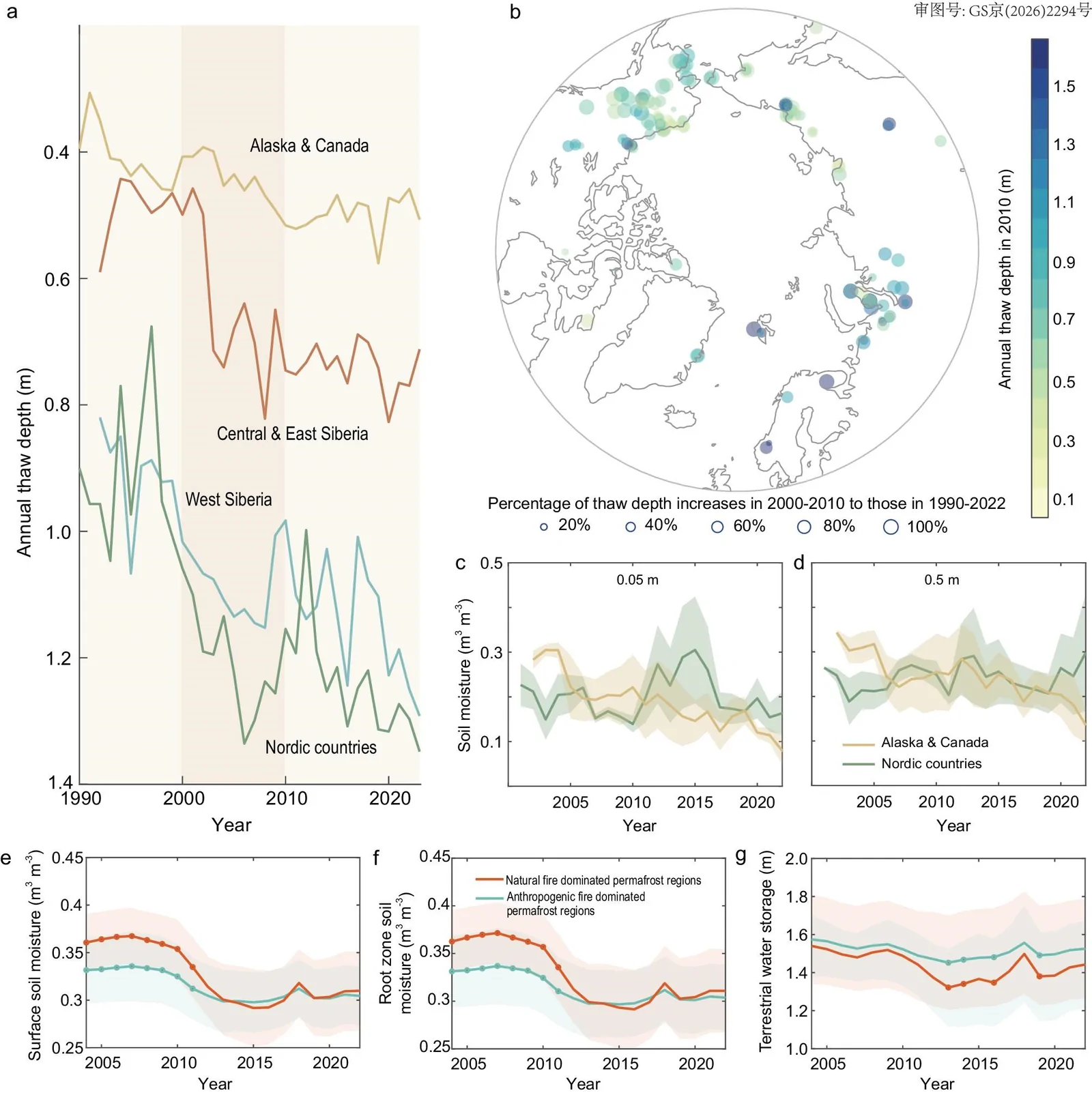
Observed changes in permafrost thermal and hydrological regimes. (a) Time series of annual thaw depth (m) in four regions of permafrost. (b) Spatial distribution of the percentage of thaw depth increases in 2000–2010 compared to those in 1990–2022. Annual thaw depth data collected from 195 stations in the Arctic-boreal area of the CALM project. (c and d) Comparison of soil moisture (m^3^m^−3^) averaged from 68 ISMN observation sites in Alaska and Northern Canada (yellow lines) and 39 ISMN observation sites in Nordic countries (green lines). The shaded area delimits the boundary of one standard deviation. Comparison of surface (e) and root zone (f) soil moisture, and terrestrial water storage (TWS; g) averaged in permafrost regions dominated by natural (red) and anthropogenic (cyan) fires. The shaded area delimits the boundary of one standard deviation, and the red and green dots indicate the statistical significance of the difference with the Monte Carlo significance test at a confidence level of 95%.

Notably, ISMN did not store any data after 2000 from sites in Siberia, preventing us from visualizing the soil moisture response to permafrost thaw for this extensive area. We therefore employed soil moisture data from the Global Land Data Assimilation System (GLDAS) [44] with terrestrial water storage (TWS) observations from assimilated Gravity Recovery and Climate Experiment (GRACE) satellite data [45] as an alternative for cross-verification. The GLDAS reanalysis data generally resembled observations regarding soil drying trend in both surface and root zone soil layers (Fig. 5e and f), with explicit soil moisture decreases happening between 2008 and 2013. In this period, the surface and root zone soil moisture in natural fire-dominated permafrost regions decreased by 18.7% and 19.4% on average, respectively. Specifically, the soil moisture decrease over natural fire-dominated regions was approximately 9% greater than that over anthropogenic fire-dominated regions. Such greater permafrost soil drying for natural fire-dominated regions in the GLDAS data corroborated our model results (Fig. 2b).

Notably, the TWS in GLDAS data for the permafrost regions dominated by natural fires also presented greater decreases compared to those dominated by anthropogenic fires, with values of ∼0.1 m lower TWS; these changes were statistically significant in years 2013, 2014, and 2016 (Fig. 5g). We posit that the lost water in shallow soil was exported from the grid cell as runoff instead of percolating downwards. Five major rivers in Siberia showed increases in streamflow after 2010 without any significant precipitation increase [46], supporting our results.

In summary, both model results and observations supported the following process. The Arctic-boreal area has warmed since 2000. The air temperature increases resulted in permafrost thaw characterized by active layer deepening and soil ice melting. The enhanced hydraulic conductivity from permafrost thaw resulted in extra water percolation and, therefore, shallow soil drying. The drier soil enhanced fuel combustibility, making a facilitated background for wildfire ignitions. The above process is pronounced only over the Arctic-boreal permafrost area, which was dominated by natural wildfires. Regarding the timeline, our model results and observations were consistent on the rapid active layer deepening over Arctic-boreal permafrost happening around 2005 and shallow soil moisture decreases happening around 2010. The timeline, therefore, was in correspondence temporally with our modeled shift in the percentage of burned area from natural fires in Arctic-boreal regions that happened around 2010.

## IMPLICATIONS AND UNCERTAINTIES

Our simulated natural and anthropogenic wildfire regimes, after involving modifications on permafrost excess ice and fire ignition parameterizations in the CLM5 model, revealed an important shift in natural wildfires over Arctic-boreal regions due to permafrost thaw. Our findings highlighted the correspondence in time between permafrost warming in the early 2000s and the increase in natural wildfires around 2010. The combination and legacy of prior warming and higher temperatures in the early 2000s collectively led to likely irreversible changes in permafrost thermal and hydrological regimes, which may be considered the precondition for natural wildfire increases. Despite some levels of spatial and temporal heterogeneities, we demonstrated that shallow soil drying dominated the hydrological impacts of permafrost thaw around the Arctic-boreal regions, starting around 2010. Compared to the modeled time of 2050 when permafrost thaw starts to enhance wildfires [12], we, in this study, spotted the evidence that this enhancement has begun around 2010. We demonstrated that the impact of current permafrost thaw has been favoring the ignition and spread of wildfires over permafrost, highlighting that permafrost thaw has become a critical driver of naturally ignited wildfires in Arctic-boreal regions.

Excess ground ice bodies in permafrost can change the thermal and hydrological responses of permafrost when thawing [22,47]. Another set of CLM5 simulations with the same fire model modifications but without permafrost excess ice produced a different timeseries of Arctic-boreal areas burned from natural fires (Fig. S15a). However, two sets of simulations were consistent on the shift around 2010 in the percentage of burned areas from natural wildfires (Fig. S15a), and the point of shift remained statistically significant by the sequential Mann-Kendall test at the confidence level of 95% (Fig. S15b). Although with different excess ice configurations, simulations agreed with each other in reproducing the accelerations of active layer deepening and shallow soil moisture decreasing over permafrost in the 2000s (Fig. 4a and Fig. S15c). In reality, there is a large uncertainty in the spatial distributions and ice contents of permafrost rich in excess ice bodies. The agreement on the timing of the shift in burned area proportion of natural wildfires validated that Arctic-boreal wildfires were expected to be enhanced as long as the active layer deepened and shallow soil dried, which had been verified through observations (Fig. 5a–d).

In this study, our model modifications on the parameterizations of both natural and anthropogenic wildfire ignitions were evaluated based on the MODIS fire occurrence observations without any disentanglement of fire ignition types. The model evaluation was based on the hypothesis that if the model could reasonably reproduce total wildfire occurrences, it also could reasonably reproduce those of natural and anthropogenic wildfires. Such a hypothesis is deficient in some way because the natural and anthropogenic wildfire distributions could be wrong simultaneously to build a reasonable total wildfire distribution, which could hardly be examined, as the observation of fire ignition type was so scarce. Nevertheless, we relied on as many additional datasets as possible for cross-verifications (Fig. S3). More importantly, the fire attributions in available datasets and our modified model results agreed that the Arctic-boreal regions, especially the Arctic-boreal permafrost regions, are dominated by naturally-ignited wildfires. In this way, we speculated that the inevitable model biases did not make our findings in this study deviate from the shift of natural wildfires in the Arctic-boreal regions.

Similar to most wildfire models, our modified fire model in the CLM5 kept using the lightning frequency climatology data without considering interannual variations as the input data for natural fire ignition sources. Since recent studies have suggested an increase in lightning activities over the Arctic-boreal region [13,48], our simulations tended to underestimate lightning-ignited fires in recent years. By importing the projected lightning frequency change as a function of temperature change developed by Janssen *et al.* [14], the ratio of burned area by lightning-ignited wildfires presents an increasing trend throughout the study period (Fig. S16a). However, the abrupt rises still exist around 2010 in the timeseries, and the abrupt change is also tested to be significant (Fig. S16b and c). Nevertheless, the model results produced both the comparable interannual variability and long-term trend of wildfire activities, suggesting that the historical variation of Arctic-boreal wildfires could still be explained primarily by soil hydrological conditions rather than lightning frequency. Therefore, the prognostic lightning parameterization scheme, especially cloud-to-ground lightning [49,50], would require the dynamic interaction of lightning and wildfires in an Earth system model for quantifying the relative contribution of increasing lightning strikes and thawing permafrost to determine Arctic-boreal wildfire regimes.

Other than the lightning frequency as the natural fire ignition source, our study was also limited by several aspects originating from model configurations. For example, the fire model did not parameterize any interactive post-fire land use land cover change (LULCC) as the dynamic vegetation model is not activated. The population density and gross domestic product (GDP), as important indicators of anthropogenic fire ignition frequency and fire suppression strength in the fire model parameterization algorithm, were also fixed with no changes through time. Such model limitations would bring biases in fire modeling, especially on the long-term predictions under climate change. Future versions of the fire model are expected to involve new processes including the vegetation recovery after fire, the time-varying population density and GDP data, potentially facilitating further developments of fire attributions.

Previous studies [2,6,7] based on site observations agreed on the facilitating effect of Arctic-boreal wildfires on permafrost degradation. Wildfire activities enhanced by permafrost thaw could, in turn, accelerate active layer deepening and shallow soil drying, in turn becoming the precondition for further wildfire enhancements. Li *et al.* [51] have highlighted the importance of the feedback process between permafrost thaw and Arctic-boreal wildfires. In this study, we complemented these findings by demonstrating that permafrost thaw has been favoring wildfires based on the land surface modeling approach, calling for a model retrieval or quantification of the permafrost-wildfire feedback. However, the current version of CLM5 and its fire model cannot simulate the responses of either soil thermal regimes or vegetation to wildfires, therefore failing to capture the feedback process. In this way, both the strength of permafrost degradation and wildfire increases [52] tend to be underestimated in this study. Further earth system model developments were found to be crucial in involving additional processes of post-fire soil and vegetation changes through developing parameterizations, allowing the dynamic interactions of wildfires with soil and vegetation for projections with less uncertainty of future climate and carbon cycle in the Arctic-boreal area.

## METHODS

We employed the latest version of the CLM5 [21] as the land surface modeling platform. The fire model developed by Li *et al.* [19] was embedded within the CLM5 by default. This process-based fire model of intermediate complexity contained separate schemes for the ignition, spread, and emission schemes of wildfires, while retaining the empirical estimates of parameters (Table 1). The detailed information on CLM model configurations and fire model modifications is available in the supplementary materials.

**Table 1.** Values of IEL and HIC chosen for the sensitivity experiments.

Parameter	Prescribed values
IEL	0.1, 0.15, 0.2, 0.22 (default), 0.25, 0.3, 0.35, 0.4, 0.45, and 0.5
HIC	0.001, 0.0025, 0.005, 0.0075, 0.01 (default), 0.0125, 0.015, 0.0175, 0.02, and 0.025

Each combination of IEL and HIC corresponded to an experiment case, with IEL = 0.22 and HIC = 0.01 set as the default values in the CLM5.

We employed multiple observations and reanalysis data for the background information on the climate and land surface conditions. The 6-hour averaged ERA5 [27] reanalysis data with a spatial resolution of 0.25 degrees and temporal coverage from 1979 to 2022 were applied as the forcing data for the land surface model. The observed annual thaw depth data were downloaded from the CALM network [41], containing the observations of annual thaw depth at 195 different sites within the Arctic-boreal area to provide comparisons to the modeled annual thaw depth. The soil moisture observational data were downloaded from the ISMN [42], collecting multi-layer soil moisture from various observational projects worldwide. This study applied data from 39 sites in Nordic countries and 68 sites in North America within the Arctic-boreal area. Since the ISMN sites differed from each other at depths at which soil moisture was observed, we linearly interpolated soil moisture observations of each site onto depths of 0.05 m and 0.5 m for consistent comparison. The daily-mean Global Land Data Assimilation System (GLDAS) [44] data with TWS assimilated from the GRACE satellite [45] were employed, offering a more observational-constrained TWS and soil moisture records as an alternative to soil moisture observations missing in Siberia.

We employed multiple satellite-based wildfire observations. Fire occurrence observation data were accessible via the Fire Information for Resource Management System (FIRMS) based on the MODIS MCD64A1 Collection 6 in the period of 2002–2022 [53–55]. The FIRMS data were re-gridded onto the model grid by calculating the summation of fire spots located in the boundary of each grid. The FIRMS data identified fire spots by the fire radiative power (FRP) value with a certain confidence level. Fire spots with a confidence level of less than 60% or FRP of less than 20 were omitted due to their potential interferences, like cloud reflections or sun glints. Only one fire spot was counted for all adjacent spots that were less than 1 km from each other in distance. Likewise, fire spots less than 1 km from each other in the two consecutive snapshots were also regarded as one single fire spot. Fire spots present longer than two weeks without any change in location (within 1 km) were discarded for their likelihood to be stationary infrared radiation sources such as thermal power plants or oil drilling platforms. As a reference for model evaluation, we applied the latest version of burned area data from GFED5 from 2002 to 2020 [23]. The information of additional datasets for model evaluation is available in the supplementary materials.

## Supplementary Material

nwag515_Supplemental_File

## Data Availability

The MODIS-based FIRMS data for fire occurrence were obtained from https://firms.modaps.eosdis.nasa.gov/download/. The CALM data were downloaded from https://www2.gwu.edu/∼calm/data/north.htm. The ISMN data are accessible through the International Soil Moisture Network (https://ismn.earth/en/dataviewer/). The ERA5 reanalysis data were distributed through the Climate Data Store (CDS; https://cds.climate.copernicus.eu/). The GFED5 burned area data are available at https://www.globalfiredata.org/data.html. The source code of the CLM5 model is freely available via https://github.com/ESCOMP/CTSM/tree/release-clm5.0.31. The supporting data for the CLM5 simulation was not only downloaded automatically by the model, but also downloaded from the NCAR server with the link https://svn-ccsm-inputdata.cgd.ucar.edu/trunk/inputdata/.
