# Supplementary material for: Permafrost thaw has enlarged the burned area proportion from naturally-ignited wildfires in Arctic-boreal regions
Source: Natl Sci Rev. 2026 Aug 20;13(17):nwag515. doi: 10.1093/nsr/nwag515 (PMC13639182; doi:10.1093/nsr/nwag515)
Supplement: Supplementary file 1 [file nwag515_supplemental_file.docx]

Supplementary materials for

**Permafrost thaw has enlarged the burned area proportion from naturally-ignited wildfires in arctic-boreal regions**

Lei Cai *et al.*

*Corresponding author. Email: [yangruowen@ynu.edu.cn](mailto:yangruowen@ynu.edu.cn)

***Additional model evaluation datasets***

Although this study focused on wildfires in the arctic-boreal regions, our model evaluation was addressed on a global scale. The fire attribution map developed by Janssen et al. [1] worked as the reference on the global scale. The fire attribution map is processed by the XGBoost machine-learning algorithm, using the observed information of lands with low human impact, the correlation between the lightning and burned area, and the correlation between the Fire Weather Index (FWI) and the burned area as the predictors to estimate natural and anthropogenic fires globally [1]. Additionally, we employed the observations of fire ignition type in scattered regions around the globe, including Canada and the United States, as well as parts of Siberia, scattered regions in Europe, and Australia. The Canadian National Fire Database [2] has the fire-point data coverage for Canada. The interagency Fire Program Analysis Fire-Occurrence Database [3] and Alaska fire-historical points database [4] have the fire-point data for the contiguous United States and Alaska. The Prometheus fire database [5] recorded the fire point data for southern France. The Forest Fire Management Victoria Agency has the fire point data for Victoria, Australia [6]. For eastern Siberia, the fire attribution is postprocessed from the remote sensing products of the MODIS MCD64A1 and the Visible Infrared Imaging Radiometer Suite VNP14IMG active fire product with an algorithm by Xu et al. [7]. The fire point observations above from separate datasets were postprocessed into gridded data by Janssen et al. [1].

***Land surface and fire model configurations***

In this study, CLM5 was configured with a grid spacing of 2.5×1.9 degrees in longitude and latitude (144×96 grid cells globally). The spatial resolution of CLM5 was coarser than that of the ERA5 forcing, and CLM5 automatically re-gridded the ERA5 data onto CLM5’s grid. Vertically, our CLM5 simulation configured a detailed soil layer structure of 49 soil layers vertically covering a depth from 0 m to 9.85 m to resolve a more detailed variation of permafrost active layer changes. The soil layers were positioned on top of 5 bedrock layers that covered a depth from 9.85 to 42 m. Note that the uniformly distributed thickness of soil layers and bedrock could be biased from reality, but it is a necessary compromise for global land surface models like CLM5. For ground ice bodies with a scale larger than the soil pore space (excess ice bodies), we applied a sub-grid scale representation of excess ground ice in the CLM5 model developed by Cai et al. [8] to retrieve a realistic response of permafrost thaw on surface thermal and hydrological conditions. The involvement of excess ice prescribed a sub-grid distribution of three different types of excess ice bodies in one single CLM5 grid cell, which simulated the melting process under the background of climate change. The melting of excess ice can lead to surface subsidence and runoff. The scenario of excess ice initialization applied three categories of excess ice in permafrost with low, medium, and high soil ice content Cai et al. [8], which was postprocessed from the 20 categories of permafrost and excess ice combinations from Brown et al. [9]. The detailed initialization of excess ice is listed in Table S1. The excess ice was initialized in all soil layers from the surface to the bottom. Then the spin-up simulation automatically melted the excess ice until the active layer.

The spin-up simulation was addressed for a period of 2000 simulation years from the prescribed climatic initial condition similar to the soil condition in 1990 (the initial condition data file was downloaded from the URL: <https://svn-ccsm-inputdata.cgd.ucar.edu/trunk/inputdata/lnd/clm2/initdata_map/clmi.I2000CLM50CRUBGC.1991-01-01.1.9x2.5_gx1v6_simyr2000_c150407.nc>). One exception is that due to the involvement of excess ground ice in permafrost regions, the initial soil temperature is set to 250 Kelvin to prevent any melting of excess ice at the first timestep. The atmospheric forcing for spin-up warms up the soil to its designated depth of active layer and retrieve a reasonable soil temperature profile. The spin-up simulation was forced by the atmospheric background cycling in 1980–1999, allowing the land surface conditions to reach their equilibrium. By the end of the spin-up period, the 20-year mean soil temperature (averaged over the period in consistency with the cycle of atmospheric forcing) for each grid at all levels barely changed (<10^-3^ ℃) for each of the 20-year periods corresponding to the cycling of atmospheric forcing. In this way, we regarded it as the completion of spin-up. The spin-up output then worked as the initial condition for both parameter calibration sensitivity runs and historical simulations. After the spin-up is done, the transient historical simulation is conducted for the years from 2000 to 2022, with the ERA5 reanalysis data as the atmospheric forcing. We determined a grid cell with or without permafrost by calculating the active layer depth based on the area-weighted soil temperature. The grid cell with the calculated active layer depth greater than 9.85 m was considered non-permafrost. The modeled permafrost extent with sub-grid excess ice involved by the end of spin-up was close to the coverage of continuous and discontinuous permafrost compared to other permafrost maps [9][10] (Figure S8). Note that the CLM5 model had a sub-tiling system to prescribe the spatial heterogeneity of land cover types [11]. The sub-grid columns of excess ice and the soil could resolve the sub-grid distributions of permafrost thermal regimes [8]. The fire model developed by Li et al. [12] was embedded within the CLM5 by default. This process-based fire model of intermediate complexity contained separate schemes for the ignition, spread, and emission schemes of wildfires, while retaining the empirical estimates of parameters. The fire model was verified for its skill in retrieving global fire patterns. For fire ignitions, the number of ignition sources $N_{i}$ was calculated as the sum of natural and anthropogenic ignitions:

$N_{i}=(I_{n}+I_{a})A_{g}$, (1)

where $I_{n}$ and $I_{a}$ are the number of natural and anthropogenic ignitions, respectively, and $A_{g}$ is the area of the grid.

The number of natural wildfire ignitions was calculated by land and atmospheric (lightning frequency) background conditions, according to the following equation that was originally developed by Prentice and Mackerras [13]:

$I_{n}=IEL\times\frac{1}{5.16+2.16cos[3\min\left( 60,\lambda\right)]}I_{l}$ , (2)

where IEL is a parameter denoted as ignition efficiency of cloud-to-ground lightning, and $I_{l}$ is the total of lightning flashes. The IEL is set to a uniform value of 0.22 for all grid cells in the original CLM5 model.

The parameterization of the human-caused fire ignitions followed that by Venevsky et al. [14], which builds a function of human ignition counts (HIC) and population density, according to the following equation:

$I_{a}=\frac{6.8\cdot HIC\cdot{D_{p}}^{0.4}}{n}$ , (3)

where $I_{\alpha}$is the human-calculated wildfire ignitions. $D_{p}$ is the population density (person per km^2^), and $n$ is the number of seconds in a month. Like the IEL, HIC is also set to be uniform with a value of 0.01 in the original CLM5 model. Natural and anthropogenic wildfire ignitions shared the same fire suppression scheme. Natural suppression was relative to the availability and combustibility of fuel, and human suppression was relative to the population density and local economy of the gross domestic product (GDP) data for each grid cell, which were available from the CLM5 input dataset and automatically downloaded from the server when running the model. After each ignition, the burned area was calculated as a function of natural parameters of wind speed, vegetation type, and fuel wetness related to relative humidity of surface atmosphere and the top 17 cm soil moisture condition, as well as the socioeconomic parameters of the population density and GDP.

Other than ordinary fires above (naturally or anthropogenically ignited), the fire model also supported fire types including deforestation fires, agriculture fires, and peat fires. Peat fires are typically characterized by smouldering combustion deep below the surface. The ignition source of peat fires is hardly detectable, and it burns with a lower temperature and over a long period of time [15]. Most peat fires in the arctic-boreal regions happen in remote areas with low human impacts. In this study, we categorized peat fires as natural fires, with agricultural and deforestation fires as anthropogenic fires. These types of fires were parameterized to export the burned area without considering ignition. We skipped the introduction of the burned area by deforestation fires, as they only occur in tropical closed forests. The fire model calculated the burned area of agricultural fires using the following equation:

$A_{b}=a_{1}f_{d}f_{e}f_{t}f_{crop}A_{g}$, (4)

where $A_{g}$ is the area of the grid cell, $a_{1}$ is a constant with the value of $1.6\times{10}^{-4}{hr}^{-1}$, $f_{t}$ is the seasonality of agricultural fires, $f_{crop}$ is the area weight of cropland in the grid cell, and $f_{d}$ and $f_{e}$ parameterize the effect of population density and GDP on burned area, which are calculated as:

$f_{d}=0.04+0.96\times\exp\left[ -\pi\left( \frac{D_{p}}{350} \right)^{0.5} \right],$	(5)

$f_{e}=0.01+0.99\times\exp\left[ -\pi\frac{GDP}{10} \right].$	(6)

Due to the above parameterization, the burned area from agricultural fires was shown to vary little from environmental changes.

Regarding peat fires, the burned area was calculated by the following equation:

$A_{b}=cf_{cli,p}f_{peat}\left( 1-f_{sat} \right)A_{g}$, (7)

where $A_{b}$ indicates the burned area fraction by peat fires, $c$ is a linear factor setting to 0.9×10^−5^ hr^−1^ for boreal peat fires by default, $f_{cli,p}$ is a function of surface wetness, which is a function of top 17 cm of soil moisture for boreal peat fires, $f_{peat}$ is the area fraction of peatland in a grid cell, and $f_{sat}$ is the area fraction of the water table at the surface or higher in a grid cell.

The lightning frequency data required by the fire model were provided as part of the CLM5 supporting data. The 3-h climatology of total lightning frequency data by NASA in 1995–2013 studies served as the ignition source of natural wildfires. The lightning data, fire occurrence data, and model simulation period were synchronized with each other for the consistency of model evaluation. The population density and GDP data were provided as the CLM5 supporting data. Likewise, this study applied the prescribed land cover distribution for the year 2000 without any land use/cover changes.

Other than the transient simulation, an idealized experiment was conducted with cycled atmospheric forcing to quantify the response of shallow soil moisture to active layer deepening without the disturbances of any interannual changes in the atmospheric forcing. Both the control and the sensitivity simulations of the experiment branched from the spin-up. The control case was forced by the cycled forcing in the year 1999 from the ERA5 reanalysis data. The sensitivity case was forced by the same forcing data, but with surface air temperature increased by 2 ℃ at each time step. Both cases were run for 100 years. The higher temperature forcing compared to the spin-up simulation would make permafrost thaw, and the constant forcing without any year-to-year changes would eventually stabilize the permafrost regime at a new equilibrium. Under this model configuration, shallow soil moisture would decrease at the beginning of the simulation in the sensitivity case because the higher temperature forcing enhanced the surface evaporation. Such a disturbance was found to stabilize after five model years, after examining the model output. We therefore conducted analyses from the 6^th^ to 100^th^ model years for the response of active layer deepening and shallow soil moisture change. We calculated the lag from active layer deepening to the negative difference in shallow-layer soil moisture in the following way. We calculated the changing rate of soil moisture response in each year as the time derivative of the soil moisture difference from the sensitivity case to the control case. For shallow-layer soil moisture, if its changing rate was lower than one standard deviation from its mean (μ-1σ) over the period, we considered it a distinctive decrease and recorded the year of happening. For deep-layer soil moisture, if its changing rate was higher than one standard deviation from its mean (μ+1σ) over the period, we considered it a distinctive increase and recorded the year of happening. The lag is defined as the difference in time from when active layer deepening to shallow-layer soil moisture decrease happened.

Regarding the possible change in lightning frequency along with the warming Arctic, the dynamic total lightning frequency data were also reconstructed based on the results by Janssen et al. [1]. The relationship between the changes in lightning frequency and temperature (% K^-1^) was processed via both the Cloud Top Height (CTH) and the Cloud Ice Flux (CIH) approaches [1], which was obtained via <https://doi.org/10.1594/PANGAEA.939352>. The lightning frequency for the years from 2000 to 2022 was then adjusted via this relationship based on the difference of the annual mean temperature in each year from that averaged from 1995 to 2013. The transient historical simulations from 2000 to 2022 were then re-run with these two lightning frequency reconstruction data imported to explore the impact of lightning frequency change in the arctic-boreal regions.

***Fire model modifications***

According to the parameterization, the occurrence of natural fires was supposed to be linearly related to the efficiency of cloud-to-ground lightning ignitions, and that of anthropogenic fire ignitions was linearly related to the number of anthropogenic ignitions per capita. The fire model, by default, configured both parameters to be uniform for all grid points, according to the fire model, though likely to vary by region in reality. In this manner, it was possible to assess the grid-scale sensitivity of wildfire occurrence to natural and anthropogenic factors to determine the dominant wildfire type for local regions. This study modified the model source code to allow for the variations of HIC and IEL on the grid scale. The model bias in the climatology of wildfire occurrence was likely to be reduced by changing the value of IEL and HIC. This study hypothesized that if the modeled climatology of total wildfire occurrence was close to the observation data, the modeled frequencies of both natural and anthropogenic wildfires were close to reality. Therefore, the model results represented reasonable numbers and ratios of natural to anthropogenic wildfires.

The modified CLM5 required a pair of IEL and HIC values for each grid. However, the ordinary parameter calibration method would lead to unaffordable computational resources in this case. This study addressed the calibration of IEL and HIC via multiple linear regression models, as the natural and anthropogenic wildfires were respectively linear functions of the lightning frequency and population density. In this study, model sensitivity experiments were designed with different sets of IEL and HIC values (Table 1).

With all possible combinations of HIC and IEL, a total of 100 sensitivity experiments were conducted in the period of 2002–2022, consistent with the temporal coverage of the MODIS-based FIRMS data. Each experiment came with a model bias field in the climatology of fire counts. For each grid point, a multiple linear regression model was built with HIC and IEL as the two independent variables and the model bias as the dependent variable, as follows:

$B=b_{0}+b_{1}\times IEL+b_{2}\times HIC$, (8)

where B is the model bias, $b_{1}$ and $b_{2}$ are the factors corresponding to IEL and HIC, respectively, and $b_{0}$ is the offset. In this equation, $b_{0}$, $b_{1}$, and $b_{2}$ were resolved and regarded as known values. The statistical significance of the constructed model was quantified by an F-test.

IEL and HIC in calculating bias did not serve as definite values. The optimal IEL and HIC combination was defined as the one that minimized the model bias. Among the two factors, IEL was a proportion with a range of 0 to 1. The HIC, however, had no upper limit in theory, but we artificially set the maximum value to 1 to avoid unrealistic conditions, causing HIC to hold the same range as IEL. Determining optimal parameters required solving a multivariable optimization problem with the function to minimize, as follows:

$f(IEL, HIC)={(b_{0}+b_{1}\times IEL+b_{2}\times HIC)}^{2}$, (9)

where $IEL\in[0,1]$ and $HIC\in[0,1]$. The constrained nonlinear optimization problem was resolved by using the sequential quadratic programming (SQP) algorithm, finding the minimum of constrained nonlinear multivariable functions. The above work was applied to all the terrestrial model grids with at least one wildfire event observed per year on average. According to the algorithm, different initial values could affect the final results of optimization. We set the initial values of IEL and HIC to their defaults (0.22 and 0.01 for all grid cells) before optimization. Tuning the initial values and reconduct the optimization for 300 times obtained the uncertainties (standard deviation) of the IEL and HIC optimization in each grid cell (Figure S1c and S1d).

The approach of building the multiple linear regression model and solving the multivariable optimization problem avoided conducting redundant test simulations that would result in unaffordable computational resources. The approach above was designed solely from the scheme of wildfire ignition sources before involving the impacts of fuel availability, combustibility, and wildfire suppression on ignition calculation.

The CLM5 model did not export anthropogenic or natural fire counts by default in addition to the total fire counts. The source code was therefore modified to export extra variables in the model output files, including the fire counts, burned area, and fire-related carbon emissions with the anthropogenic and natural causes. These exported variables could quantify the number of anthropogenic and natural wildfire events and their respective impacts. After all calibration and modification work, the calibrated HIC and IEL values for each grid were imported into the model, conducting a historical offline simulation forced by the ERA5 reanalysis data from 2000 to 2022. All analyses in the modeled wildfire and permafrost variabilities were based on the results of this model case.

***Significance test for detecting changing points***

In this study, we applied the sequential Mann-Kendall test to detect the point of abrupt changes in the time series. The Mann-Kendall mutation test branched from the Mann-Kendall method of time series [16]. The Mann-Kendall method served as a rank-based non-parametric significance test for detecting monotonic trends, and it did not require a normal distribution of the sample to be tested. The sequential Mann-Kendall test was originally proposed by Sneyers [17] as a technical note published by the World Meteorological Organization. It was specifically designed to detect the point of abrupt change of autocorrelated time series, like meteorological and hydrological data. The sequential Mann-Kendall test has been commonly used in detecting the point of shift in climatological data [18][19].

For a timeseries with a length of $n$, $d_{k}$ is computed as:

$d_{k}=\sum_{k=1}^{n-1} \sum_{j=k+1}^{n} sgn\left( x_{j}-x_{k} \right)$, (10)

in which	$sgn\left( x_{j}-x_{k} \right)=\left\{ \begin{aligned} +1 x_{j}-x_{k}>0 \\ 0 x_{j}-x_{k}=0 \\ -1 x_{j}-x_{k}<0 \end{aligned} \right.$	(11)

The calculation returned the number of positive differences minus that of negative differences.

The forward sequence of the time series, UF, could be calculated as:

$UF_{k}=\frac{d_{k}-E(d_{k})}{\sqrt{Var(d_{k})}} (k=1,2,\ldots\ldots n)$, (12)

where $E(d_{k})$ and $Var(d_{k})$ are the mean value and variance being calculated by

$\left\{ \begin{aligned} E\left( d_{k} \right)=\frac{k\left( k-1 \right)}{4} \\ Var\left( d_{k} \right)=\frac{k\left( k-1 \right)\left( 2k+5 \right)}{72} \end{aligned} \right.$	(13)

For detecting the point where abrupt change occurs, a backward sequence UB, which is in the reverse order of the timeseries UF, can be calculated as

$UB_{k}=-UF_{k^{'}} (k=n+1-k^{'})$. (14)

By taking the 95% confidence level, the threshold for UF and UB was ±1.96. With the curves of UF and UB drawn, if an intersecting point of UF and UB was present within ±1.96, an abrupt change occurred after the intersecting moment and was tested as significant.

***Results for grid-scale parameter calibrations and evaluations***

Addressing the multivariable optimization problem determines the optimal HIC and IEL values for each grid (Figure S1a and S1b), with their standard deviations when selecting different initial values from which the optimization starts (Figure S1c and S1d). However, a high value of parameters does not guarantee a high number of wildfire occurrences will be modeled for a certain grid. Instead, it implies that fire occurrence in a certain grid cell will be sensitive to lightning frequency (for natural wildfires) or population density (for anthropogenic wildfires). As a result, both HIC and IEL have shown spatial variations with higher-than-default HIC and IEL found in the northern parts of North America, northern Asia, and the majority of Europe, Africa, and South America. These two parameters with grid-scale variation were imported into the CLM5 for model evaluation simulation. After modification, the spatial distribution of the modeled annual fire counts climatology was significantly closer to the results (Figure S2a-c). Large biases were widely spread around the globe, modeled by the original CLM5 (Figure S2d), while the biases were mostly cut off after modification (Figure S2e). The pattern correlation coefficient (PCC) increased significantly from 0.58 to 0.90, and the RMSE decreased from over 7000 to below 4000.

Comparing our fire attribution results (Figure S3a) to observations (Figure S3a) and the global map by Janssen et al. [1] (Figure S3b), our study generated a similar attribution pattern in general. The bias in the percentage of burned area by naturally ignited fires existed mostly on the boundary of regions with natural and anthropogenic fires dominated (Fig. S3c and S3d). This meant that the two datasets were congruent in major regions with dominant types of fires. Specifically for the arctic-boreal regions, there were few grid points with the mismatch of dominant fire types, most of which were in Alaska, where our model suggested a domination of anthropogenic fires (Figure S3e and S3f). The cause of wildfires in Alaska remains under debate. Nevertheless, our conclusion regarding the shift in natural fires was even more pronounced if the increase in wildfires in Alaska were considered to be naturally dominated. In this manner, we speculated that this bias did not invalidate the main conclusions of this study.

**Figure S1. Spatial distributions of the HIC and IEL and their uncertainties.** The spatial patterns of the calibrated IEL (a) and HIC (b). Black lines on color bars in (a) and (b) indicate the default values of HIC and IEL originally set by the fire model. The spatial patterns of the standard deviations of the calibrated IEL (c) and HIC (d), the standard deviations are calculated from reconducting the optimization with different initial IEL and HIC values.

**Figure S2 Model evaluation with MODIS fire counts data as a reference.** Annual climatology of fire counts in (a) FIRMS observation data, (b) original CLM5 model, and (c) modified CLM5 model. (d) Bias in annual fire counts (times year^-1^) before modification. (e) Bias in annual fire counts (times year^-1^) after modification. Black dots in (e) indicate grid cells with 10% or smaller biases in annual mean fire counts modeled by the modified CLM5.

**Figure S3. The model evaluation of fire attribution.** Percentage of burned area from natural fires in (a) observations in key regions around the globe and (b) from the dataset by Janssen et al. [1] from the machine learning technique. Biases in the percentage of burned area from natural fires with (c) observations in key regions worldwide and (d) from the dataset obtained by Janssen et al. [1]. Grid cells with a mismatch of dominated fire type from our model and from (e) observations in key regions worldwide, and (f) from the dataset obtained by Janssen et al. [1] Grid cells modeled as dominated by natural fires, while also observed to be dominated by anthropogenic fire, are marked green. Grid cells that were modeled as dominated by anthropogenic fires, while observed to be dominated by natural fire, are marked in brown.

**Figure S4 The fire attribution maps presenting the boundary of uncertainties**. (a) The fire attribution map with the calibrated IEL plus one standard deviation and HIC minus one standard deviation. (b) The fire attribution map with the calibrated IEL minus one standard deviation and HIC plus one standard deviation.

**Figure S5. Burned area climatology from 2002 to 2022 in (a) the GFED5 data and (b) the modified CLM5 model.**

**Figure S6. Spatial patterns of burned area change.** Changes in the annual mean burned area between 2011-2022 and 2000-2010 in (a) the GFED5 data and (b) the CLM5 model. Relative changes in the annual mean burned area between 2011-2022 and 2000-2010 in (c) the GFED5 data and (d) the CLM5 model.

**Figure S7. Spatial patterns of changes in annual mean burned area with the two types of fires.** CLM5-modeled Changes in the annual mean burned area between 2011-2022 and 2000-2010 of (a) natural fires and (b) anthropogenic fires. Relative changes in the annual mean burned area between 2011-2022 and 2000-2010 of (a) natural fires and (b) anthropogenic fires.

 **Figure S8 Spatial patterns of fire season atmospheric condition changes in the ERA5 reanalysis data.** Changes in (a) surface air temperature and (b) precipitation. The fire season in each year is defined by the period with 0-17 cm soil temperature greater than 0℃.

**Figure S9. Temporal variations of temperature (℃) and precipitation (mm/year) from 2000 to 2022 in the arctic-boreal area (60–90°N).** The temperature and precipitation were obtained that ERA5 reanalysis data that forced the CLM5 simulations.

**Figure S10. Grid cells with permafrost modeled by the CLM5 and the corresponding annual thaw depth (m) by the end of the spin-up period.**

**Figure S11. Temporal statistics of wildfires over permafrost.** (a) Percentage (%) of burned area over permafrost in the arctic-boreal regions. (b) Sequential Mann-Kendall test result on the timeseries of burned area proportion from wildfires over permafrost. UF (red line) and UB (blue line) serve as the two statistical parameters calculated from forward and backward, respectively. The intersections represent the specific year(s) after which the time series abruptly changed. The interaction lying between the two black dashed lines indicated that the point of abrupt change passed the 95% confidence level.

**Figure S12. Statistics of the average burned area for each wildfire event in the Arctic-Boreal regions in terms of permafrost regimes and wildfire ignition types.**

**Figure S13. Responses and lags of active layer deepening and soil moisture change in the idealized experiment.** The time series of the differences from the sensitivity case to the control case in (a) active layer depth (m), 0-0.5 m soil moisture (m^3^/m^3^), and 0.5-9.85 m soil moisture (m^3^/m^3^) for the grid cell located at 127.5°E, 63.5°N. The active layer depth is calculated as the annual maximum of thaw depth in a year. The soil moisture and burned area are averaged over the fire season in each year. The thickened lines in (b) and (c) indicate the distinctive changes in soil moisture deviating by one standard deviation or more based on the mean of the derivatives of the time series. (d) The burned area (km^2^/month) averaged over summer in the grid cell. The gray shading indicates that the first 5 years are excluded from screening lags. The lags from the responses of active layer deepening and 0-0.5 m soil moisture change in all grid cells within the Arctic-boreal permafrost to calculate the probability density functions of (e) active layer depth and (f) annual mean surface air temperature (℃) in the sensitivity case measured at the time when the corresponding soil moisture decrease happened.

**Figure S14. Soil moisture anomalies at depths of 0–9.85 m compared to the last year of spin-up for the soil layers.**

**Figure S15. Arctic-boreal wildfire regimes modeled by the CLM5 with fire model modification, but without involving excess ice.** (a) Time series of the percentage of burned area (%) from fires on permafrost (orange line) and from natural fires (red line). (b) The sequential Mann-Kendall test results on the time series of burned area proportion by natural fires. UF (red line) and UB (blue line) serve as the two statistical parameters calculated forward and backward, respectively. The intersections represent the specific year(s) after which the time series abruptly changed. The interaction lying between the two black dashed lines indicated that the point of abrupt change passed the 95% confidence level. (c) Soil moisture anomalies at a depth of 0–8 m compared to the last year of spin-up for the soil layers. Anomalies are calculated with the last year of spin-up as a reference for each corresponding soil layer. The black line indicates the annual thaw depth from 2000 to 2022, averaged over the arctic-boreal permafrost.

**Figure S16. Temporal statistics of arctic-boreal wildfires with the changing frequency of lightning.** (a) Percentage (%) of burned area over permafrost in the arctic-boreal regions. The model configuration is the same as in Figure 1b, but with the lightning frequency changing as a function of temperature change according to the study by Janssen et al. [1]. The lightning projections are based on the approaches of Cloud Top Height (CTH) and Cloud Ice Flux (CIF). Sequential Mann-Kendall test results on the time series of burned area proportion from wildfires in (a) with the (b) CTH and (c) CIF approach in reconstructing lightning frequencies. UF (red line) and UB (blue line) serve as the two statistical parameters calculated from forward and backward, respectively. The intersections represent the specific year(s) after which the time series abruptly changed. The interaction lying between the two black dashed lines indicated that the point of abrupt change passed the 95% confidence level.

| The applied permafrost type according to Brown’s permafrost map | Overall excess ice content | Area weights for sub-grid excess ice categories |
| --- | --- | --- |
| clf; clf; slf; ilf; clr; dlr; slr; ilr | 5% | 20 % low |
| cmf; dmf; smf; imf; dhr; shr; ihr | 15% | 20 % low; 22 % medium |
| chr | 15% | 20 % low; 14 % high |
| dhf; shf; ihf | 25% | 20 % low; 22 % medium; 14 % high |
| chf | 25% | 20 % low; 28 % high |

**Table S1 Prescribed area weights of different excess ice tiles for each type of permafrost.** For each permafrost type, the first letter indicates the permafrost extent. “c” stands for continuous permafrost, “d” stands for discontinuous permafrost, “s” stands for sporadic permafrost, and “i” stands for isolated permafrost. The second letter indicates the excess ice content. “h” stands for high ice content, “m” stands for medium ice content, and “l” stands for low ice content. The third letter indicates the terrain and overburden. “f” stands for the landscapes of lowlands, highlands, and intra-land intermontane depressions, and “r” stands for the landscapes of mountains, highlands, ridges, and plateaus. The naming and categorization of permafrost types strictly follow the permafrost map by Brown et al. [9]. The volumetric contents of excess ice for low, medium, and high excess ice tiles in the CLM5 model are set to 0.25 m^3^/m^3^, 0.45 m^3^/m^3^, and 0.7 m^3^/m^3^, following the initialization scheme by Cai et al. [8].

**Reference**

[1] Janssen TAJ, Jones MW, Finney D *et al.* Extratropical forests increasingly at risk due to lightning fires. *Nat Geosci 2023*; **16**: 1136–1144.
[2] Stocks BJ, Mason JA, Todd JB *et al.* Large forest fires in Canada, 1959–1997. *J Geophys Res-Atmos 2002;* **107**: 8149.
[3] Short KC. A spatial database of wildfires in the United States, 1992-2011. *Earth Syst Sci Data 2014*; **6**: 1-27.
[4] Kasischke ES, Williams D, Barry D. Analysis of the patterns of large fires in the boreal forest region of Alaska. *Int J Wildland Fire 2002*; **11**: 131–144.
[5] Ganteaume A, Guerra F. Explaining the spatio-seasonal variation of fires by their causes: The case of southeastern France. *Appl. Geogr 2018;* **90**: 69–81.
[6] Nampak H, Love P, Fox-Hughes P *et al*. Characterizing Spatial and Temporal Variability of Lightning Activity Associated with Wildfire over Tasmania, Australia. *Fire 2021*; **4**: 10.
[7] Xu W, Scholten RC, Hessilt TD *et al.* Overwintering fires rising in eastern Siberia. *Environ Res Lett 2022;* **17**: 045005.
[8] Cai L, Lee H, Aas KS *et al*. Projecting circum-Arctic excess-ground-ice melt with a sub-grid representation in the Community Land Model. *The Cryosphere 2020*; **14**: 4611–4626.
[9] Brown J, Ferrians O, Heginbottom JA *et al.*  Circum-Arctic Map of Permafrost and Ground-Ice Conditions. (GGD318, Version 2). [Data Set]. Boulder, Colorado, USA. *National Snow and Ice Data Center 2002*; Date Accessed 10-25-2025.
[10] Obu J, Westermann S, Bartsch A *et al*. Northern Hemisphere permafrost map based on TTOP modelling for 2000–2016 at 1 km^2^ scale. *Earth-Sci Rev 2019*, **193**: 299–316.
[11] Lawrence DM, Fisher RA, Koven CD *et al*. The Community Land Model Version 5: Description of New Features, Benchmarking, and Impact of Forcing Uncertainty. *Journal of Advances in Modeling Earth Systems 2019*; **11**: 4245–4287.
[12] Li F, Zeng XD, Levis S. A process-based fire parameterization of intermediate complexity in a Dynamic Global Vegetation Model. *Biogeosciences 2012;* **9**: 2761–2780.
[13] Prentice SA, Mackerras D. The ratio of cloud to cloud-ground lightning flashes in thunderstorms, *J Appl Meteorol 1977;* **16**: 545–550.
[14] Venevsky S, Thonicke K, Sitch S *et al.* Simulating fire regimes in human-dominated ecosystems: Iberian Peninsula case study, *Glob. Change Biol. 2002*; **8**: 984–998.
[15] Kohlenberg AJ, Turetsky MR, Thompson DK. Controls on boreal peat combustion and resulting emissions of carbon and mercury. *Environ Res Lett 2018;* **13**: 035005.
[16] Mann HB. Nonparametric tests against trend. *Econometrica 1945*; **13**: 245–259.
[17] Sneyers R. On the Statistical Analysis of Series of Observations. *Technical Note No. 143, WMO 1990;* No. 725 415: 192pp.
[18] Wang J. Determining the most accurate program for the Mann-Kendall method in detecting climate mutation. *Theor Appl Climatol 2020*; **142**: 847-854.
[19] Wang W, Yi Z, Chen D. Mann-kendall mutation analysis of temporal variation of apparent stress in Qinba mountains and its adjacent areas. *IOP conference series: earth and environmental science 2021;* **660**: No. 1.
